# Prevalence and correlates of meeting the Canadian 24-hour movement guidelines among a sample of Canadian parents during the COVID-19 pandemic

**DOI:** 10.1186/s44167-023-00027-3

**Published:** 2023-09-05

**Authors:** Scott Rollo, Abigail Sckrapnick, Julie E Campbell, Sarah A Moore, Guy Faulkner, Mark S Tremblay

**Affiliations:** 1 Healthy Active Living and Obesity Research Group, Children’s Hospital of Eastern Ontario Research Institute, 401 Smyth Road, Ottawa, ON K1H 8L1 Canada; 2 School of Epidemiology and Public Health, Faculty of Medicine, University of Ottawa, Ottawa, ON K1H 8M5 Canada; 3 School of Health and Human Performance, Dalhousie University, Halifax, NS B3H 4R2 Canada; 4 School of Kinesiology, University of British Columbia, Vancouver, BC V6T 1Z1 Canada; 5 Department of Pediatrics, Faculty of Medicine, University of Ottawa, Ottawa, ON K1H 8L1 Canada; 6 Department of Health Sciences, Carleton University, Ottawa, ON K1S 5B6 Canada

**Keywords:** Physical activity, Sedentary behaviour, Sleep, Movement behaviours, Parents, Public health recommendations, COVID-19

## Abstract

**Background:**

Parents’ own movement behaviours can influence those of their children, thus contributing to the health and well-being of the whole family. Parents experienced a shift in work and childcare responsibilities during the COVID-19 pandemic. This may have led to a reduction in their healthy movements. This study examined the prevalence and correlates of meeting vs. not meeting the individual and combined recommendations within the Canadian 24-hour movement guidelines for adults among a sample of Canadian parents during the second wave (October 2020) of the COVID-19 pandemic.

**Methods:**

Parents of children aged 5–17 years (n = 1,477) responded to a cross-sectional survey conducted in October 2020. A total of 21 self-reported correlates, including parental and child demographics, and change in family movement behaviours/characteristics were assessed. Parental movement behaviours were reported and classified as meeting or not meeting each of the guidelines. Associations between correlates and meeting each of the guidelines were examined using multiple logistic regression.

**Results:**

The proportion of parents who met the moderate-vigorous physical activity (MVPA), recreational screen time, sleep duration and combined guidelines were 21.2, 51.0, 66.1, and 9.1%, respectively. Being a parent ≥ 45 years old, having a university education, and higher levels of outdoor play were associated with meeting the combined guidelines. Age, dwelling type, family hobbies, and outdoor play were associated with meeting the MVPA recommendation. Employment status, education level, dog ownership, children’s age, family physical activity, and levels of distress were associated with meeting the recreational screen time recommendation. Geographical region, dwelling type, and levels of distress were associated with meeting the sleep duration recommendation.

**Conclusions:**

Few Canadian parents were meeting the combined 24-hour movement guidelines recommendations for MVPA, recreational screen time, and sleep six months into the COVID-19 pandemic. Several socio-demographic, behavioural, and COVID-19-related factors emerged as significant correlates of meeting vs. not meeting the individual and/or combined recommendations within the guidelines. The findings provide various avenues for which to target future movement behaviour interventions and guideline adoption for parents.

## Introduction

Canada’s *24-hour movement guidelines for adults (aged 18–64 years)* provide evidence-based recommendations for moderate­-to-vigorous physical activity (MVPA; ≥150 min/week), sedentary behaviour (SB; ≤8 h/day, including ≤ 3 h/day of recreational screen time) and sleep (7 to 9 h/day) [1]. A healthy 24-hour movement profile, comprised of greater physical activity (PA), less SB and sufficient sleep, is associated with a decreased risk of all-cause mortality and numerous non-communicable diseases including cardiovascular disease, type 2 diabetes, several types of cancer, depression, and anxiety disorders [2–6]. Using a representative sample of Canadian adults, a recent study using pre-COVID-19 data found that meeting the integrated 24-hour movement guidelines and different combinations of recommendations within the guidelines was associated with a number of favourable health indicators [7].

To promote health and well-being among Canadian families during the ongoing COVID-19 pandemic, it is important to gain timely descriptive information on the proportion of parents meeting both the individual (i.e., MVPA, SB [recreational screen time], sleep) and combined (i.e., all three movement behaviours) 24-hour movement guideline recommendations, and to comprehensively assess how adherence to these recommendations varies across specific socio-demographic and family characteristics of people living in Canada. The movement behaviours of parents are important to assess when it comes to the movement of the whole family. Beyond just material support and encouragement of positive movement behaviours for their children, parents play an important role in modelling behaviours to their children, such that parent movement behaviours such as MVPA, sedentary time, and screen time have been found to be positively correlated with those of their children [8, 9]. Further, parents are a demographic that generally obtain less physical activity than other Canadian adults without children [10, 11], and there is a general lack of research available regarding the correlates of parental sleep and sedentary behaviour [9]. For these reasons combined, the 24-hour movement behaviours of parents are important to study and subsequently develop interventions for, in order to support the health of families as a whole.

It has now been more than three years since the World Health Organization (WHO) declared the COVID-19 virus outbreak a global pandemic on March 11, 2020, due to the risks of acute respiratory disease posed by the SARS-CoV-2 virus [12, 13]. Given that the COVID-19 virus is transmitted through respiratory droplets and contact routes [12], the outbreak led to states of emergency, community-wide lockdowns, and stay-at-home orders, as well as the introduction of policies and guidelines to encourage physical distancing and slow the spread of COVID-19, which have significantly impacted the daily routines of Canadian adults and their families [14]. In Canada, the restrictions and public health measures throughout the first year of the pandemic included practicing physical distancing from others by at least two metres (except for those living in your immediate household), requiring people to wear a face mask in indoor public places, using caution in closed spaces/crowded places, practicing hand hygiene/respiratory etiquette, prohibiting community and social gatherings, cancelling team sports and related events, and closing playgrounds and parks (in some jurisdictions) [15, 16]. In addition, there have been repeated periodic closures of non-essential businesses, public/leisure facilities, offices/workplaces, and schools in response to various waves of the pandemic [15]. After a late spring and summer of lower COVID-19 case counts, in October 2020 specifically, cases were on the rise again, at approximately 2,000 cases per day in Canada, warranting the declaration of a second wave of COVID-19 [17]. Although case counts were rising throughout the country, the fall restrictions were not as strict as in the initial wave, and many parks, outdoor recreation spaces, sport facilities, and schools were open (albeit with new regulations and protocols varying by region); thus, generally providing increased opportunities for physical activity and favourable movement behaviours compared to early in the pandemic [18, 19]. As a result of ever-changing work-from-home policies and in-person learning being replaced by homeschooling and online learning, parents have been repeatedly challenged to adapt family practices and lifestyle routines during this pandemic [20]. Early in the pandemic, children were learning from home, structured activities were ceased – parents played a critical role in supporting their child’s education and activities, while often also maintaining their own work-related responsibilities. With this substantial increase in workload, it would be assumed that parents had less time to engage in their own physical activities.

Despite the known benefits of regular PA and a healthy lifestyle as prime modalities for the prevention of numerous non-communicable diseases [2], very little mention has been made as to the potential benefits of maintaining a healthy active lifestyle throughout the COVID-19 pandemic. In fact, several of the imposed precautions and restrictions have worked against individuals’ healthy lifestyle habits. This is unfortunate as PA has also been shown to be a protective factor against COVID-19. Regular MVPA has been shown to have a positive effect on the immune system, response to vaccination, and risk of community-acquired infectious disease incidence and mortality [21]. Among a sample of 48,440 adults diagnosed with COVID-19, consistently meeting PA guidelines was strongly associated with a reduced risk for severe COVID-19 outcomes among patients, including a lower risk of hospitalization, admission to the intensive care unit, and death [22]. Importantly, Colley et al. [23] found that maintaining opportunities for outdoor exercise and limiting screen time may promote better mental and general health during periods of confinement among Canadian adults. Therefore, promoting healthy movement behaviours throughout the remainder of the pandemic, and in future pandemics should be a priority.

Unfortunately, negative effects on movement behaviours have been shown as a result of the COVID-19 virus outbreak. A systematic review investigated differences in PA and SB before vs. during the COVID-19 lockdown and found the majority of studies reported decreases in PA and increases in SB during lockdown periods as a result of COVID-19 among children and youth [24]. In two large-scale national studies, Moore and colleagues found that only 2.6% and 3.1% of children and youth living in Canada were meeting the combined 24-hour movement behaviour guidelines during the first (April 2020) and second (October 2020) waves of the COVID-19 pandemic, respectively [18, 25]. Furthermore, specific factors were associated with guideline adherence during the pandemic [18, 25–27]. For instance, Guerrero et al. [26] identified different profiles of children and youth who were more and less likely to meet the Canadian 24-hour movement guidelines, based on specific demographic, behavioural, social, and environmental characteristics. COVID-19 policies and restrictions, which vary by province, have also led to regional differences in outdoor play among children and youth and families across Canada [19, 28, 29]. The negative consequences of the COVID-19 pandemic on the movement and play behaviours of children and youth are not unique to Canada and have been shown in several countries to varying degrees [30–33].

The COVID-19 pandemic has had a negative impact on adults’ health and movement behaviours in a number of countries [24, 34]. The majority of studies have reported that PA decreased and SB increased during COVID-19 lockdown periods compared to pre-COVID-19 [24]. However, evidence pertaining to how the 24-hour movement behaviours of Canadian adults, and more specifically parents, have been impacted is less clear. Specific to PA levels, findings from three Canadian studies suggest that PA decreased among Canadian adults during the initial months of the pandemic [35–37]. Utilizing data from the 2018 and 2020 cycles of the Canadian Community Health Survey, another study found there was no significant change in the percentage of adults aged 18 to 64 years who reported meeting the Canadian MVPA recommendation during the COVID-19 pandemic (September-December 2020) compared to pre-pandemic (October-December 2018) [38].

Currently, the prevalence of Canadian adults meeting the new 24-hour movement guideline recommendations, both individually and in combination, during the COVID-19 pandemic is unknown. For purposes of surveillance and monitoring, it is important to explore social-ecological factors associated with meeting vs. not meeting the movement behaviour guidelines during the COVID-19 pandemic. A number of studies have examined social-ecological correlates associated with adherence to 24-hour movement guidelines in the early years and children and youth both prior to and during the COVID-19 pandemic [27, 28, 39, 40]. The influence of social-ecological correlates, including demographic, behavioural, social, and environmental characteristics, on individual movement behaviours of adults has been established [41–44]; however, there is limited research on correlates of meeting vs. not meeting the combined movement behaviour guidelines [45, 46]. More specifically, information on parents’ movement behaviours throughout the COVID-19 pandemic have not been studied. Parents are a population of adults who often face additional barriers in meeting the guidelines [10, 11], and these reasons may have been particularly exacerbated by the pandemic. This information could be used to support efforts for guideline implementation and adoption, and to inform and target the development of programs and policies to encourage and support healthy movement behaviours among different segments of the Canadian adult population that may be particularly vulnerable to not meeting the guideline recommendations during the pandemic. Therefore, the purpose of this study was to examine the prevalence and correlates of meeting vs. not meeting the individual and combined recommendations within the Canadian 24-hour movement guidelines for adults among a representative sample of parents living in Canada during the second wave of the COVID-19 pandemic. Given the changing routines of families and increased demands on families, we hypothesized that low proportions of parents would be meeting the 24-hour movement recommendations. We hypothesized that younger parental age, higher parental education, and lower parental distress would be positively related to healthy movement behaviours. We further hypothesized that living in dwellings in mixed land-use areas and dog ownership may also be positively related to healthy movement behaviours of parents. These socio-demographic factors are commonly associated with movement behaviours and we expect that they play important roles during the pandemic as well.

## Methods

### Study design and population

This study used data from a cross-sectional survey conducted in October 2020 by ParticipACTION [47], a national non-profit organization that promotes physical activity and healthy movement behaviours among Canadians. The purpose of this repeated, cross-sectional national survey was to assess the *ongoing* impact of the COVID-19 pandemic on the movement and active play behaviours (i.e., PA, SB, screen time, sleep, and outdoor play) of Canadian children and youth aged 5–17 years and their parents. ParticipACTION conducted the first survey in April 2020 [25] and a follow-up survey in October 2020 [18], which was extended to include reporting of parental movement behaviours after this was identified as important in our qualitative work [48]. The survey was previously reported to have good reliability [25].

Recruitment was conducted by a third-party market research company, Maru/Matchbox, that has an online consumer database of more than 120,000 Canadian panelists. Maru/Marchbox panelists are comparable with the Canadian census in terms of age, gender, region, income, employment, and language spoken. Panelists are recruited through email invitation and website sign-up and receive small cash incentives ($0.50-$3.00 CDN) for completing surveys. Because of the ability to rapidly recruit large, representative samples, this sampling strategy is commonly used by ParticipACTION and other national organizations and researchers (e.g., [9, 25]). Maru/Matchbox panelists consent to participate in research when they sign up for the panel.

For the 6-month follow-up survey, a sample of parents (or adult caregivers) of Canadian children (aged 5–11 years) and/or youth (aged 12–17 years) were invited to complete the brief online survey (in English or French) during the survey timeline (October 15–20, 2020), approximately 6 months after COVID-19 was declared a global pandemic [13] and at the beginning of the second wave of the virus in Canada [49].

### Survey and survey administration

The survey was developed utilizing a socioecological framework to consider variables at the child, family, and community levels [50, 51]. Briefly, the contents of the survey addressed socio-demographic characteristics, child movement behaviours, and parent movement behaviours. Potential respondents were sent an email link to the survey. Respondents passively consented to participate when they agreed to complete the survey which took approximately 15 min to complete. If a parent had more than 1 child, they were asked to respond based on their child whose given first name came first alphabetically. Respondents completed a series of screening questions and were excluded if anyone in the household had been diagnosed with COVID-19 in the last month or if they were presently under self-isolation (i.e., quarantine) orders. Collected data were cleaned by Maru/Matchbox and received by ParticipACTION. Secondary use of data was approved by Dalhousie University’s Research Ethics Board (#2020–5351). Data verification was completed by the study’s authors prior to data analysis. For a more detailed description of the ParticipACTION survey, please see Moore et al. [25] and Moore et al. [18].

### Dependent variables

The survey consisted of four questions that assessed parent’s 24-hour movement behaviours. These were adapted from the Canadian Health Measures Survey [52] and included: *(1) “In the last week, how many hours did you usually spend sleeping in a 24-hour period (including naps but excluding time spent resting)?”, (2) “In the last week, how many hours did you usually spend sedentary (e.g., sitting or reclining) in a 24-hour period?”, (3) “In the last week, how many hours did you usually spend in front of screens during your leisure time in a 24-hour period (e.g., watching TV and videos, playing video games, browsing the web, texting, etc.)?”, and (4) “Throughout the last week, how much time did you spend engaging in moderate-to-vigorous physical activity (e.g., if you have walked 20 minutes on 4 days, please report 80 minutes)?”.* The response options for the first three questions ranged from 0 to 16 h; whereas the last question was reported in minutes.

#### Meeting the 24-Hour guidelines

Parents were considered to have met the 24-hour movement guidelines if they met the time-specific recommendations for MVPA, SB (recreational screen time) and sleep duration [1]. Adherence to the MVPA recommendation (≥ 150 min per week) was determined if the weekly minutes of MVPA reported by the respondent was ≥ 150 min per week. The reference group was those not meeting the recommendation. A recommendation of ≤ 3 h per day of recreational screen time is included as a subcomponent of the 24-hour movement guidelines SB recommendation (i.e., required within the sedentary time recommendation). Screen time was categorized as a binary variable based on meeting (vs. not meeting) the recommended no more than three hours per day of recreational screen time. The reference group was those not meeting the recommendation. Sleep duration was coded as a binary variable with those not meeting age-specific recommendations (7–9 h/day) used as the reference group.

### Correlates

A total of 21 self-reported variables categorized as parental socio-demographic factors (n = 9), child socio-demographic factors (n = 3) and change in family characteristics and movement/play behaviours as a result of the COVID-19 outbreak (n = 9) were examined as potential correlates. Parental socio-demographic factors included age (18–44 years or ≥ 45 years), gender (man or woman), geographical region (Western provinces, Ontario, Quebec, Atlantic provinces), marital status (married or single), employment status (employed or not employed), highest level of education (secondary school or less, college, university, post-graduate studies), dwelling type (apartment, house, other), dog ownership (yes or no), and number of children (1, 2, 3+). Child socio-demographic factors included the child’s age (child [5–11 years] or youth [12–17 years]), gender (boy or girl), and disability status (yes or no). Variables related to changes in family characteristics and movement/play behaviours as a result of the COVID-19 outbreak included child schooling (in-person, blended learning [a mixture of in-person at school and online], online/virtual learning, not attending), primary playmate of child (parent or other), family time spent in PA (less vs. same or more), family time spent in SB (less vs. same or more), time spent in outdoor play or engaging in MVPA with child (less vs. same or more), new family hobbies (yes vs. no), family use of online resources/applications to support healthy movement behaviours (yes vs. no), deterioration of child’s health (e.g., existing condition worsened or new condition developed) (yes vs. no), and family distress (0–5 vs. 6–10 [higher rating indicates higher distress]).

### Statistical analysis

Survey participants who provided responses outside the range of pre-determined cut-off values that were established for each movement behaviour (MVPA > 4200 min/week; sleep duration < 4 or > 20 h/day; SB > 20 h/day; and recreational screen time > 20 h/day) were excluded from the analyses.

Descriptive statistics were used to calculate frequencies and proportions (categorical variables) or means and standard deviations (SD) (continuous variables) for all independent and dependent variables. Initially, univariate (unadjusted) logistic regression models were used to assess the associations between each correlate and meeting the combined and individual 24-hour movement guideline recommendations. Associations between specific correlates and meeting each of the MVPA, recreational screen time, and sleep recommendations separately and combined were then examined using multiple logistic regression. Specifically, each model included all correlates that were significant predictors in the unadjusted univariate models. Odds ratios and their 95% CIs were reported for all models. Results were considered statistically significant at *p* < 0.05 (two-sided test) if the 95% CIs did not cross 1.0. The Hosmer-Lemeshow and Nagelkerke pseudo-R^2^ test statistics were used to assess the goodness of fit for each regression model. Analyses were conducted in SPSS version 27.0 (IBM, United States).

## Results

A total of 1,653 parents of children aged 5 to 17 years completed the survey in October 2020. Participants were excluded when child age was not entered and when parental age was implausible (*n* = 85; e.g., parental age 20 years while child age 15 years). Only one participant reported residing in the territories and was therefore removed. Participants who provided implausible responses for movement behaviours (n = 2 for MVPA, n = 15 for SB, n = 9 for screen time, n = 64 for sleep duration) were also excluded from the analyses. The final sample comprised 1,477 parents. Parent and child (descriptive statistics) characteristics are presented in Table 1. The majority of respondents were aged 18–44 years (57.9%), women (58.8%), employed (80.4%), married or cohabiting (79.9%), and college or university graduates (86.6%). The mean nightly sleep duration and daily recreational screen time were 7.3 h and 4.6 h, respectively. The mean weekly MVPA was 112.0 min. The proportion of parents who met the MVPA, recreational screen time, and sleep duration guidelines were 21.2, 51.0, and 66.1%, respectively. Overall, only 9.1% of parents were meeting the combined 24-hour movement guideline recommendations (MVPA, SB [recreational screen time], and sleep).

**Table 1:** Participant characteristics

Variables	Full sample (n = 1,477)
**Parent socio-demographic variables**	
Age group	
18–44 years	855 (57.9)
≥45 years	622 (42.1)
Sex	
Male	609 (41.2)
Female	868 (58.8)
Geographical region	
Western provinces	448 (30.3)
Ontario	573 (38.8)
Quebec	238 (16.1)
Atlantic provinces	218 (14.8)
Marital status	
Married	1,180 (79.9)
Single	297 (20.1)
Employment status	
Employed	1,188 (80.4)
Unemployed	289 (19.6)
Education	
Secondary school or less	198 (13.4)
College	482 (32.6)
University	542 (36.7)
Post-graduate studies	255 (17.3)
Dwelling type	
Apartment	201 (13.6)
House	1,260 (85.3)
Other	16 (1.1)
Dog ownership	
No	889 (60.2)
Yes	588 (39.8)
# of children	
1	715 (48.4)
2	587 (39.7)
≥3	175 (11.9)
**Child socio-demographic variables**	
Child age	
5–11 years	677 (45.8)
12–17 years	800 (54.2)
Child gender	
Boy	746 (50.5)
Girl	716 (48.5)
Child disability status	
Yes	136 (9.2)
No	1,341 (90.8)
Change in family characteristics and movement/play behaviours variables	
Child schooling	
In-person	964 (65.3)
Blended	231 (15.6)
Home	282 (19.1)
Child’s primary playmate	
Other	1,231 (83.3)
Parent	246 (16.7)
Family PA	
Less	438 (29.7)
Same or more	1,039 (70.3)
Family SB	
Less	127 (8.6)
Same or more	1,350 (91.4)
Outdoor play	
Less	249 (16.9)
Same or more	1,228 (83.1)
Family hobbies	
No	1,297 (87.8)
Yes	180 (12.2)
Use of online resources	
No	1,338 (90.6)
Yes	139 (9.4)
Child health condition	
No	1,411 (95.5)
Yes	66 (4.5)
Family distress	
0–5	1,119 (75.8)
6–10	358 (24.2)
24-hour movement behaviours	
Weekly MVPA, min/week	112.0 (262.3)
Daily recreational screen time, h/day	4.6 (3.6)
Daily sleep duration, h/day	7.3 (1.7)

MVPA = moderate- to vigorous-intensity physical activity; PA = physical activity; SB = sedentary behaviour. Data presented as mean (standard deviation) for continuous variables and frequencies (percentages) for categorical variables

Note: For child gender, 15 parents reported that their child did not identify as a ‘boy’ or ‘girl’ or preferred not to say

Table 2 shows the univariate associations between each of the self-reported correlates and meeting both the individual and combined 24-hour movement guideline recommendations. The adjusted odds ratios and 95% CIs for the associations of the self-reported correlates with meeting the individual and combined guidelines among Canadian parents are presented in Tables 3, 4, 5 and 6. Age (adults aged ≥ 45 years: 1.7 [1.2, 2.4]), higher education (university education: 3.3 [1.5, 7.0]; post-graduate studies: 3.1 [1.4, 7.1]), and greater time spent in outdoor play with a child (same or more: 2.7 [1.4, 5.1]) were significantly associated with a greater likelihood of meeting the combined guidelines.

**Table 2:** Univariate associations between correlates and adherence to individual and combined movement behaviour recommendations

Variables	MVPA	Screen Time	Sleep	All
Parent socio-demographic variables	OR (95% CI)	OR (95% CI)	OR (95% CI)	OR (95% CI)
Age group (ref = 18–44 years)				
≥45 years	**1.5 (1.2, 1.9)**	0.9 (0.7, 1.0)	1.0 (0.8, 1.3)	**1.7 (1.2, 2.4)**
Sex (ref = Male)				
Female	1.2 (1.0, 1.6)	1.2 (0.9, 1.4)	0.9 (0.8, 1.2)	1.1 (0.8, 1.6)
Geographical region (ref = Western provinces)				
Ontario	0.8 (0.6, 1.1)	0.8 (0.6, 1.0)	**0.7 (0.6, 1.0)**	0.7 (0.5, 1.1)
Quebec	0.7 (0.5, 1.1)	0.9 (0.7, 1.3)	1.0 (0.7, 1.5)	0.7 (0.4, 1.2)
Atlantic provinces	0.7 (0.5, 1.1)	0.8 (0.6, 1.1)	0.7 (0.5, 1.0)	0.7 (0.4, 1.2)
Marital status (ref = Married)				
Single	0.8 (0.6, 1.2)	0.8 (0.6, 1.0)	**0.7 (0.6, 0.9)**	0.9 (0.6, 1.4)
Employment status (ref = Employed)				
Unemployed	1.0 (0.7, 1.3)	**0.6 (0.5, 0.8)**	**0.7 (0.5, 0.9)**	0.9 (0.6, 1.4)
Education (ref = Secondary school or less)				
College	1.1 (0.7, 1.7)	1.1 (0.8, 1.6)	0.9 (0.7, 1.3)	1.7 (0.8, 3.8)
University	1.3 (0.8, 1.9)	**1.5 (1.1, 2.1)**	**1.5 (1.1, 2.1)**	**3.2 (1.5, 6.8)**
Post-graduate studies	1.7 (1.1, 2.7)	**1.6 (1.1, 2.3)**	1.4 (1.0, 2.1)	**3.2 (1.4, 7.1)**
Dwelling type (ref = Apartment)				
House	**1.7 (1.1, 2.6)**	1.2 (0.9, 1.6)	**1.6 (1.1, 2.1)**	1.7 (0.9, 3.1)
Other	0.4 (0.1, 3.1)	1.1 (0.4, 3.2)	1.6 (0.6, 4.9)	1.1 (0.1, 8.6)
Dog ownership (ref = No)				
Yes	1.0 (0.7, 1.2)	**0.8 (0.7, 1.0)**	1.1 (0.9, 1.4)	0.9 (0.7, 1.4)
# of children (ref = 1)				
2	1.2 (0.9, 1.5)	1.1 (0.9, 1.4)	1.0 (0.8, 1.3)	1.0 (0.7, 1.5)
≥3	1.6 (1.1, 2.3)	1.2 (0.9, 1.7)	1.0 (0.7, 1.4)	1.6 (1.0, 2.7)
Child socio-demographic variables				
Child age (ref = 5–11 years)				
12–17 years	1.2 (1.0, 1.6)	**0.8 (0.6, 1.0)**	1.0 (0.8, 1.3)	1.3 (0.9, 1.9)
Child gender (ref = Boy)				
Girl	1.0 (0.8, 1.3)	1.1 (0.9, 1.3)	1.2 (0.9, 1.5)	1.3 (0.9, 1.9)
Child disability status (ref = Yes)				
No	1.0 (0.6, 1.5)	1.2 (0.8, 1.6)	**1.7 (1.2, 2.5)**	1.7 (0.8, 3.5)
Change in family characteristics and movement/play behaviours variables				
Child schooling (ref = In-person)				
Blended	1.2 (0.8, 1.7)	0.8 (0.6, 1.1)	1.3 (1.0, 1.8)	1.4 (0.9, 2.3)
Home	1.0 (0.7, 1.3)	0.9 (0.7, 1.1)	0.8 (0.6, 1.1)	0.9 (0.6, 1.5)
Child’s primary playmate (ref = Other)				
Parent	0.7 (0.5, 1.0)	0.8 (0.6, 1.0)	1.2 (0.9, 1.7)	**0.6 (0.3, 1.0)**
Family PA (ref = Less)				
Same or more	1.2 (0.9, 1.5)	**1.5 (1.2, 1.8)**	1.1 (0.9, 1.4)	1.4 (0.9, 2.1)
Family SB (ref = Less)				
Same or more	1.5 (0.9, 2.4)	1.3 (0.9, 1.9)	1.3 (0.9, 1.9)	1.1 (0.6, 2.0)
Outdoor play (ref = Less)				
Same or more	**2.2 (1.5, 3.3)**	**1.4 (1.1, 1.9)**	1.2 (0.9, 1.6)	**2.4 (1.3, 4.6)**
Family hobbies (ref = No)				
Yes	**1.5 (1.1, 2.2)**	1.0 (0.7, 1.3)	0.8 (0.6, 1.2)	1.4 (0.8, 2.2)
Use of online resources (ref = No)				
Yes	1.5 (1.0, 2.2)	1.1 (0.8, 1.5)	1.0 (0.7, 1.5)	1.3 (0.8, 2.3)
Child health condition (ref = No)				
Yes	1.0 (0.5, 1.8)	0.7 (0.4, 1.1)	**0.5 (0.3, 0.9)**	1.0 (0.4, 2.3)
Family distress (ref = 0–5)				
6–10	0.9 (0.6, 1.2)	**0.7 (0.6, 0.9)**	**0.6 (0.4, 0.7)**	0.7 (0.4, 1.0)

OR (95% CI) = odds ratio (95% Confidence Intervals); MVPA = moderate- to vigorous-intensity physical activity; PA = physical activity; SB = sedentary behaviour. Statistically significant associations (95% CIs were significant at the *p* < 0.05 level if they did not cross 1.0) are highlighted in bold

**Table 3:** Adjusted associations of correlates with adherence to the combined movement behaviour recommendations

Variables	All three recommendations
	OR (95% CI)
Age group (ref = 18–44 years)	
≥45 years	**1.7 (1.2, 2.4)**
Education (ref = Secondary school or less)	
College	1.7 (0.8, 3.7)
University	**3.3 (1.5, 7.0)**
Post-graduate studies	**3.1 (1.4, 7.1)**
Child’s primary playmate (ref = Other)	
Parent	0.6 (0.3, 1.0)
Outdoor play (ref = Less)	
Same or more	**2.7 (1.4, 5.1)**

OR (95% CI) = odds ratio (95% Confidence Intervals). Statistically significant associations (95% CIs were significant at the *p* < 0.05 level if they did not cross 1.0) are highlighted in bold. Hosmer-Lemeshow test statistic: *p* > 0.05; Nagelkerke pseudo-R^2^ = 0.06

**Table 4:** Adjusted associations of correlates with adherence to the MVPA recommendation

Variables	MVPA
	OR (95% CI)
Age group (ref = 18–44 years)	
≥45 years	**1.5 (1.2, 1.9)**
Dwelling type (ref = Apartment)	
House	**1.6 (1.1, 2.5)**
Other	0.4 (0.1, 3.2)
Outdoor play (ref = Less)	
Same or more	**2.2 (1.4, 3.2)**
Family hobbies (ref = No)	
Yes	**1.5 (1.1, 2.2)**

OR (95% CI) = odds ratio (95% Confidence Intervals); MVPA = moderate- to vigorous-intensity physical activity. Statistically significant associations (95% CIs were significant at the *p* < 0.05 level if they did not cross 1.0) are highlighted in bold. Hosmer-Lemeshow test statistic: *p* > 0.05; Nagelkerke pseudo-R^2^ = 0.04

**Table 5:** Adjusted associations of correlates with adherence to the recreational screen time recommendation

Variables	Screen time
	OR (95% CI)
Education (ref = Secondary school or less)	
College	1.1 (0.8, 1.5)
University	**1.5 (1.0, 2.0)**
Post-graduate studies	**1.5 (1.0, 2.2)**
Employment status (ref = Employed)	
Unemployed	**0.6 (0.5, 0.8)**
Child age (ref = 5–11 years)	
12–17 years	**0.8 (0.6, 1.0)**
Outdoor play (ref = Less)	
Same or more	1.2 (0.9, 1.7)
Family distress (ref = 0–5)	
6–10	**0.8 (0.6, 1.0)**
Family PA (ref = Less)	
Same or more	**1.4 (1.1, 1.8)**
Dog ownership (ref = No)	
Yes	**0.8 (0.6, 1.0)**

OR (95% CI) = odds ratio (95% Confidence Intervals); PA = physical activity. Statistically significant associations (95% CIs were significant at the *p* < 0.05 level if they did not cross 1.0) are highlighted in bold. Hosmer-Lemeshow test statistic: *p* > 0.05; Nagelkerke pseudo-R^2^ = 0.05

**Table 6:** Adjusted associations of correlates with adherence to the sleep duration recommendation

Variables	Sleep
	OR (95% CI)
Education (ref = Secondary school or less)	
College	0.9 (0.7, 1.3)
University	1.4 (1.0, 2.0)
Post-graduate studies	1.4 (0.9, 2.1)
Geographical region (ref = Western provinces)	
Ontario	**0.7 (0.6, 1.0)**
Quebec	1.1 (0.8, 1.6)
Atlantic provinces	0.8 (0.5, 1.1)
Marital status (ref = Married)	
Single	0.8 (0.6, 1.1)
Dwelling type (ref = Apartment)	
House	**1.5 (1.1, 2.1)**
Other	1.9 (0.6, 5.8)
Employment status (ref = Employed)	
Unemployed	0.8 (0.6, 1.1)
Child schooling (ref = In-person)	
Blended	1.3 (1.0, 1.8)
Home	0.9 (0.7, 1.2)
Family distress (ref = 0–5)	
6–10	**0.6 (0.5, 0.8)**
Child disability status (ref = Yes)	
No	1.4 (1.0, 2.0)
Child health condition (ref = No)	
Yes	0.6 (0.4, 1.1)

OR (95% CI) = odds ratio (95% Confidence Intervals). Statistically significant associations (95% CIs were significant at the *p* < 0.05 level if they did not cross 1.0) are highlighted in bold. Hosmer-Lemeshow test statistic: *p* > 0.05; Nagelkerke pseudo-R^2^ = 0.07

Age (adults aged ≥ 45 years: 1.5 [1.2, 1.9]), dwelling type (house: 1.6 [1.1, 2.5]), new family hobbies (yes: 1.5 [1.1, 2.2]), and greater time spent in outdoor play with a child (same or more: 2.2 [1.4, 3.2]) were significantly associated with a greater likelihood of meeting the MVPA recommendation.

Having a higher education (university education: 1.5 [1.0, 2.0]; post-graduate studies: 1.5 [1.0, 2.2]) and engaging in higher levels of family PA (same or more: 1.4 [1.1, 1.8]) were significantly associated with a greater likelihood of meeting the recreational screen time recommendation; whereas unemployment (0.6 [0.5, 0.8]), higher levels of family distress (6–10: 0.8 [0.6, 1.0]), having an older child (aged 12–17 years: 0.8 [0.6, 1.0]), and dog ownership (0.8 [0.6, 1.0]) were significantly associated with a lower likelihood of meeting the recreational screen time recommendation.

Dwelling type (house: 1.5 [1.1, 2.1]) was significantly associated with a greater likelihood of meeting the sleep duration recommendation; whereas higher levels of family distress (6–10: 0.6 [0.5, 0.8]) was significantly associated with a lower likelihood of meeting the sleep duration recommendation. Compared to living in the Western provinces, those who resided in Ontario (0.7 [0.6, 1.0]) had a significantly lower likelihood of meeting the recommendation for sleep duration. No other geographical differences were observed.

## Discussion

We examined the association between several socio-demographic and family characteristic factors and meeting the Canadian 24-hour movement guidelines in a large sample of Canadian parents during the second wave of the COVID-19 pandemic. To our knowledge, this was the first study to examine the prevalence of guideline adherence and identify important correlates associated with meeting vs. not meeting the individual and combined Canadian 24-hour movement guidelines among Canadian parents during the COVID-19 pandemic.

Numerous studies have reported that the COVID-19 pandemic and accompanying restrictions have had detrimental effects on individual movement behaviours [24, 30, 34, 53, 54]. Specific to Canadian adults, it has been shown that levels of PA decreased early in the pandemic [35–37]. To date, no studies have explored levels of adherence to the adult 24-hour movement guidelines during the COVID-19 pandemic. Previous studies found that the proportion of Canadian adults who met the overall 24-hour movement guideline recommendations, in their entirety, was low (~ 7–9%) [7, 45, 46]; however, these studies were all conducted pre-COVID and using datasets prior to the release of the guidelines [1]. In the current study, only 21.2, 51.0, 66.1, and 9.1% of parents were meeting the MVPA, recreational screen time, sleep duration, and all three recommendations, respectively, during the second wave of the COVID-19 pandemic. The extent to which the COVID-19 pandemic and related public health measures (e.g., social distancing, “stay-at-home” orders, work from home policies, closures of schools) were negatively associated with 24-hour movement guideline adherence among adults (and specifically parents), is unknown. Yet, studies have shown that the percentage of children and youth meeting the overall 24-hour movement guidelines has declined as a result of the pandemic [30]. Studies that examine temporal trends and changes in the movement behaviours of parents over the remainder of the pandemic and throughout recovery are warranted. Whether there are differences between the proportions of Canadian parents versus the general adult population meeting the 24-hour movement guideline recommendations during this pandemic also needs to be established.

Findings indicated that parents aged 45 years or older and those who engaged in greater levels of outdoor play with their children were significantly more likely to meet both the individual MVPA recommendation and combined guideline recommendations. Traditionally, research has shown that younger adults display healthier levels of movement behaviours than older adults [42–45]; however, in our survey, it was found that, parents aged ≥ 45 years were more likely to have older children; thus, the children may be less dependent on them in terms of care and supervision. During the COVID-19 pandemic, when faced with daily challenges (e.g., work-from-home policies; home-based online schooling), it is plausible that older parents had more time (and energy) to focus on their own health and well-being. Intuitively, it makes sense that parents who spent the same or more time playing outdoors with their children (since the COVID-19 outbreak and related restrictions) were more active, less sedentary, and perhaps, more cognizant of the importance of sufficient sleep. This finding is also congruent with previous research that has shown increases in outdoor PA/sport since the outbreak began and parental support factors (e.g., encouragement, engagement, co-participation) to be positively associated with Canadian children and youth’s adherence to the 24-hour movement guidelines during the COVID-19 pandemic [18, 25–27].

Parents with a higher level of education were significantly more likely to meet both the individual recreational screen time recommendation and combined guideline recommendations. Before the COVID-19 outbreak, only a few studies explored the association between education level and 24-hour movement guideline adherence and findings were inconsistent. For instance, Rollo et al. [45] reported no significant association between household education level and meeting the combined guideline or SB recommendations among Canadian adults, but having a higher education was associated with meeting the MVPA recommendation. Similarly, Weatherson et al. [46] found that Canadian university students with parents with higher education were more likely to meet the overall guidelines. Among Latin American adults, those with a higher level of education had a lower likelihood of meeting both the individual screen time and combined recommendations [55]. Studies that have examined the relationship between level of education and 24-hour movement guideline adherence among adults post-COVID-19 are scarce; however, Rhodes et al. [41] found that during the COVID-19 pandemic, inactive Canadian adults had less formal education than their active counterparts who were meeting the MVPA guideline.

Parents whose primary residence was an apartment or condo were significantly less likely to meet the individual recommendations for MVPA and sleep duration. This finding is consistent with those of previous studies examining levels of movement behaviours among children and youth during the COVID-19 pandemic that found children and youth living in houses spent more time outdoors engaged in PA compared to those living in apartments [18, 25, 39]. A study of toddlers and preschoolers in Chile during the pandemic also found that living in an apartment, and even small dwelling size in general, were strong predictors of declines in the children’s PA and sleep quality, which could very well be similar factors affecting the parents’ movement behaviours [56]. While the dwelling type of a family cannot easily be changed, providing safe spaces to engage in PA outside the home during these circumstances, thus also benefitting sleep duration and quality, is essential.

Finally, parents who reported higher levels of family distress were significantly less likely to meet the individual recommendations for recreational screen time and sleep duration. Similar to our findings, Werneck et al. [57] observed that clustering of unhealthy movement behaviours was associated with poorer mental health among Brazilian adults during the pandemic. Recent evidence has suggested that sleep problems during COVID-19 may be attributable to the stress caused by dramatic changes in daily life (e.g., home confinement, reduced social interaction, lower levels of PA), worsening mental health (e.g., worry, depression, anxiety, loneliness), and being in isolation/quarantine [54]. In line with our findings, previous studies that examined 24-hour movement behaviours among children and youth since the onset of COVID-19 have found that those with more anxious parents were less likely to meet the PA and screen time recommendations [58].

Other factors that emerged as significant correlates of meeting individual guideline recommendations included new family hobbies and/or activities as a result of the COVID-19 outbreak and related restrictions (MVPA), greater family time spent in PA (screen time) and being employed (screen time). Finally, parents residing in Ontario had a significantly lower likelihood of meeting the sleep duration recommendation than those living in the Western provinces. There could be various reasons for this difference, with one potential explanation being that family distress in Ontario was reported as second highest in Canada (M (SD) = 4.75 (2.67)), behind Manitoba (M (SD) = 5.06 (2.59)), among survey participants. The stress that families endured from all the changes such as working from home, home-schooling children, majorly reducing their social interactions, working under stressful situations, financial insecurity, and dealing with ongoing health risks, especially in a province such as Ontario, that experienced some of the highest case-counts, can all negatively impact their functioning and sleep [59]. Contrarily, there is also a bi-directional relationship between distress and sleep, where perhaps the new daily routines of parents during COVID-19 in high-risk provinces such as Ontario have led to new duties to fulfill, leaving them with fewer hours in the day to sleep, in turn leading to higher family distress [59]. No matter the true relationship, the unprecedented circumstances during these times impacted the sleep of parents.

Our findings suggest that the proportion of Canadian parents of school-aged children who met the combined recommendations for MVPA, recreational screen time, and sleep duration, within the new 24-hour movement guidelines, was very low. Moreover, the findings suggest that several socio-demographic factors and family characteristics (including parental age, education level, dwelling type, outdoor play, and levels of distress) are associated with meeting the guidelines and may influence parents’ movement behaviours during the COVID-19 pandemic. Relative to the benefits, education about the benefits of a healthy lifestyle and advice to maintain healthy levels of movement behaviours has never received the attention it deserves, and this has been especially brought to light during the pandemic. We argue that through the recovery process, in the event of continued waves of infections, and in preparing for future public health challenges, public health measures and policy decisions should consider ways to preserve, support, and encourage healthy levels of movement behaviours for Canadian adults/parents and their families. The findings herein provide important information for both local- and national-level efforts to promote and create the spaces and assistance required to achieve healthy movement behaviours among Canadian parents during and beyond the on-going COVID-19 pandemic. Additionally, this study provides information that will allow for international and cross-cultural comparisons of adherence to 24-hour movement guidelines among adults, specifically parents, and their correlates and determinants during the SARS-CoV-2 virus outbreak.

### Strengths and Limitations

This is the first study to identify several correlates associated with meeting the Canadian 24-hour movement guidelines and the individual recommendations for MVPA, recreational screen time and sleep among parents of children and youth (ages 5 to 17 years) living in Canada during the COVID-19 pandemic. We assessed a wide range of socio-demographic factors and family characteristics. Given that the new Canadian 24-hour movement guidelines for adults were released in October 2020, this paper gives the insight as to whether or not Canadian parents were meeting these guidelines during the pandemic, and potential implications for their children observing or not observing these healthy behaviours.

It is also worth noting the limitations associated with this study. First, the repeated cross-sectional design of this study prevents causal inferences from being made regarding the associations observed (and the first survey [25] did not assess parental movement behaviours) and the extent to which COVID-19 impacted parents’ movement behaviours. Future longitudinal studies that examine temporal trends in meeting vs. not meeting the 24-hour movement guidelines according to relevant socio-demographic factors are needed to evaluate the longer-term consequences of the COVID-19 virus outbreak and recovery on the movement behaviours of parents. Second, 24-hour movement behaviour data were self-reported with single-item measures; hence, measurement error, including recall bias, social desirability, and potential over-/under-estimation, associated with these subjective measures may have influenced our findings. The single-item measures may have also limited construct variability and inhibited the ability to examine intra-individual variability. Third, we also recognize that slightly more parents who identified as female completed the survey (59%) compared with parents who identified as male (41%), and this may have biased results as there are differing responsibilities amongst mothers and fathers [60]. Pandemic parenting may have also been different for mothers compared with fathers [61]. Further studies may wish to purposefully recruit parents who identify as male. We also recognize, a fourth limitation, that the survey was only offered in English and French. While 98.2% of people living in Canada speak either language [62], we have not represented those that do not speak English or French. A fifth limitation is that we measured ST and not all sedentary behaviours more completely, such that other types of sedentary time and their associated correlates may also provide interesting findings related to parent movement behaviours.

## Conclusion

In this cross-sectional survey study, we found that only 9% of Canadian parents were meeting the combined 24-hour movement guideline recommendations for MVPA, recreational screen time, and sleep during the second wave of the COVID-19 pandemic. 21 socio-demographic, family characteristic, and behavioural factors were associated with meeting vs. not meeting the individual and/or combined recommendations within the guidelines. Specifically, parents who are younger (aged 18–44 years), less educated, live in an apartment/condo, and report higher levels of family distress may be particularly vulnerable for not meeting the recommendations within the 24-hour movement guidelines. Creating policies for communities and workplaces that support parents to be able to engage in favourable movement behaviours is imperative. Building accessible spaces to carry-out physical activities, even in times of elevated public health measures, providing education on and encouraging the importance of family PA and parents engaging in outdoor play with their children, and ensuring adequate supports are in place for families in times of uncertainty and stress may be feasible avenues for increasing levels of adherence to the guideline recommendations [48] and to support the health of families as a whole. Large-scale interventions and public health promotion efforts are required to encourage healthy levels of daily movement behaviours and improve compliance with the 24-hour movement guidelines among Canadian parents during the on-going COVID-19 pandemic and through the recovery process.

## Data Availability

This dataset is available from ParticipACTION upon reasonable request and completion of a data transfer agreement.

## List of Abbreviations

MVPA: Moderate-vigorous physical activity
SB: Sedentary behaviour
PA: Physical activity
WHO: World Health Organization
SD: Standard deviation
